# Prevalence and predictors of hypovitaminosis D among the elderly in subtropical region

**DOI:** 10.1371/journal.pone.0181063

**Published:** 2017-07-31

**Authors:** Chi-Hsien Huang, Yu-Tung Anton Huang, Yu-Cheng Lai, Cheuk-Kwan Sun

**Affiliations:** 1 Department of Family Medicine, E-Da Hospital, Kaohsiung City, Taiwan; 2 Center for Evidence-based Medicine, E-Da Hospital, Kaohsiung City, Taiwan; 3 School of Medicine for International Students, I-Shou University, Kaohsiung City, Taiwan; 4 Master Program of Long-Term Care in Aging, College of Nursing, Kaohsiung Medical University, Kaohsiung City, Taiwan; 5 Adjunct Research Fellow, Department of Medical Research, Kaohsiung Medical University, Kaohsiung City, Taiwan; 6 Adjunct Research Fellow, Department of Medical Research, China Medical University Hospital, Taichung City, Taiwan; 7 Department of Emergency Medicine, E-Da Hospital, Kaohsiung City, Taiwan; Medical University Innsbruck, AUSTRIA

## Abstract

The prevalence of low vitamin D status in the elderly population of subtropical area and the potential risk/protective factors have not been addressed. This cross-sectional questionnaire-based study, which collected demographic/anthropometric data and information on diet habit and sun exposure, recruited 170 subjects with mean age 70.9±5.6 in rural areas of southern Taiwan. Serum 25-OH vitamin D, calcium, and intact parathyroid hormone were also measured. Using cut-off level of 30 ng/mL, subjects were divided into low (n = 95) and normal (n = 75) serum vitamin D groups. The results demonstrated a low vitamin D status in 30.6% of men and 57.7% of women. Dietary vitamin D intake was another factor associated with vitamin D status (p = 0.02). Logistic regression identified inadequate intake of vitamin D-rich food as the only risk factor for low vitamin D status in men (OR = 4.55, p = 0.01), whereas inadequate sun exposure was the only predictable risk with dose-response relationship in women (low vs. high sun exposure, OR = 6.84, p = 0.018; moderate vs. high sun exposure, OR = 6.67, p = 0.005). In conclusion, low vitamin D status was common in the elderly of subtropical rural areas. Low sun exposure and inadequate dietary vitamin D consumption were associated with a low vitamin D status in females and males, respectively.

## Introduction

Vitamin D, which is one essential nutrient for intestinal calcium absorption to facilitate mineralization of bone [1], not only helps in maintaining muscle strength and function [2], but has also been reported to reinforce immunity [3, 4] as well as improve outcomes of cardiovascular diseases [5–7]. At the other end of the spectrum, vitamin D deficiency has been shown to be associated with rickets, osteomalacia, osteoporosis, risk of fracture, and cancers [1, 8–10]. It has also been reported to be associated with colorectal, lung, prostate, breast and ovarian cancer [11]. Consistently, vitamin D supplementation has been reported to reduce all-cause cancer mortality [12]. Moreover, vitamin D supplementation seems to lower the risk of acute respiratory infection and asthma exacerbation [13, 14].

Vitamin D can be produced by the skin through exposure to ultraviolet-B (UVB) or obtained from the diet. UVB converts 7-dehydrocholesterol to pre-vitamin D3 in the skin, followed by conversion to vitamin D3. On the other hand, vitamin D2 (ergocalciferol) is acquired exclusively from diet. Vitamin D (i.e., vitamin D2 and D3) is metabolized in the liver to 25-hydroxyvitamin D (25-(OH)D), which is often used as a biomarker to determine vitamin D status [15]. Although vitamin D production has been found to be adequate as long as there is sunlight exposure of arms and legs for 5 to 30 minutes twice weekly [16, 17], low vitamin D status remains a public health problem worldwide [18].

Although there is no definite consensus so far, vitamin D deficiency and insufficiency are usually defined as 25-hydroxyvitamin D < 20 ng/mL and 20–30 ng/mL, respectively [1, 2, 19–21]. According to large-scale cross-sectional studies in North America, one-third of all subjects was demonstrated to have 25-(OH)D level below 20 ng/mL, two-thirds of those below 30 ng/mL [22, 23]. In other words, low vitamin D status is a common condition in western populations. On the other hand, in addition to the dearth of study on the prevalence of low vitamin D status in Asia [18, 24, 25], there is no investigation addressing the issue in subtropical countries. Moreover, the target population in previous studies focused mainly on adolescents and young adults rather than the elderly whose population has been on the increase worldwide. Indeed, aging is known to be associated with decreased 7-dehydrocholesterol concentration in the skin, resulting in reduction of vitamin D3 production [17, 26, 27]. Interestingly, although previous studies carried out across different countries in Southern Asia revealed a high prevalence of low vitamin D status, no particular attention has been paid to the elderly population [28, 29]. Although risk factors for a low vitamin D status (i.e., age, gender, race, body mass index, latitude, season, nutritional intake, sun exposure, and physical activity) have been reported for the general population [23, 29–31], risk and protective factors contributing to the vitamin D status in the elderly have not be addressed. The purposes of the study, therefore, is to determine the prevalence of low vitamin D status in the elderly population living in a subtropical area and to explore the potential risk or protective factors.

## Subjects and methods

### Study population

Healthy volunteers aged 65 or older were randomly recruited from a tertiary referral hospital in a rural area in Southern Taiwan from February to August 2015. Community dwellers older than 65 years with written informed consent were eligible for the present study, whereas those with active cancer in previous 5 years, end-stage renal disease, liver cirrhosis, psychiatric disease, or disorders known to affect bone mineral metabolism including hyperthyroidism, hyperparathyroidism, osteomalacia, Crohn's disease, cystic fibrosis, or Paget’s disease were excluded.

### Study design and parameters

This is a prospective cross-sectional study based on information acquired through questionnaires and blood samples. After signing the informed consent, each subject was required to complete a questionnaire for collecting information on age, gender, body height, body weight, past history, medical and drug history, status of vitamin D supplement, status of sun exposure, and consumption of vitamin D-rich food through a food diary. According to the total diet scores, the participants were classified into those with high vitamin D intake and those with relatively low intake. Blood samples were obtained from all subjects for the quantification of 25-hydroxyvitamin D (25-(OH)D), intact parathyroid hormone (iPTH), and serum calcium at the central laboratory of the institute. The whole study protocol was reviewed and approved by the Institutional Review Board of E-Da Hospital (EMRP-102-045). Informed consents were obtained from all subjects.

### Questionnaire design

The instrument used for assessing the factors affecting an individual’s vitamin D status was a questionnaire containing four major items, including (1) basic demographic, anthropometric, and clinical information including medical history, drug history (2) consumption of vitamin D supplements, including type and dosage (3) extent of sun exposure and habits of sunscreen use (4) food frequency questionnaire (FFQ) regarding the consumption of vitamin D-rich food. To estimate the amount of vitamin D from food, a food frequency questionnaire (FFQ) was developed by an experienced dietician (S1 and S2 Tables). The vitamin D contents of the commonly available vitamin D-rich food items included in the questionnaire are summarized in supporting information S3 Table [1, 32]. In each item, consumption was recorded by frequency per week, which was transformed into scores (i.e., never = 0 point, 1–2 times/week = 1 point, more than 2 times/week = 2 points). The sum of total scores was used to estimate the intake during recent 3 months. Physical activity was measured by using Taiwanese version of the International Physical Activity Questionnaire, which has been shown to be a valid and reliable tool [33].

### Blood tests

Five milliliters of blood was obtained from each subject between 10 a.m. and 11 a.m. in the morning. All subjects were refrained from calcium-containing food and beverage including calcium supplement tablets, and milk as well as caffeine- or alcohol-containing beverage for at least 12 hours before collection of blood samples. Serum levels of 25-hydroxyvitamin D (25-(OH)D), intact parathyroid hormone (iPTH) were determined at the central laboratory of the institute with standard methods using chemiluminescent immunoassay (Abbott ARCHITECT i2000, Illinois, U.S.A.). Serum calcium level was determined by o-cresolphthalein complexone (OCPC) method (Toshiba C, Tokyo, Japan). All analyses were completed within 12 hours of blood sample harvesting.

### Definitions

Degree of sun exposure was divided into three categories according to the duration between 8 a.m. and 5 p.m.: Low (<1 hour/week), medium (1–2 hours/week), and high (>2 hours/week). Sun screen use was defined as the use of any sun screen products within 3 months prior to the study regardless of the frequency. Vitamin D supplements were defined as all oral supplements containing vitamin D other than food. A diet scoring system was developed based on the frequency of consumption of vitamin D-rich food products including fish, sardine, herring, salmon, oysters, eggs, fortified cereals, fortified soymilk, and fortified milk. The testing subjects were categorized into low (score 0—5) and high (score 6—10) diet scorers. The four seasons were defined as: Spring (March—May), summer (June—August), fall (September—November), and winter (December—February). Three levels of Physical activity, which were proposed by the scoring protocol in guidelines for data processing and analysis of the Taiwan version of International Physical Activity Questionnaire (IPAQ), were used to label participants as low, moderate, and high groups[33]. The definition of vitamin D deficiency and insufficiency are usually defined as 25-hydroxyvitamin D < 20ng/mL and 20-30ng/mL, respectively [1]. The testing subjects in our study were divided into low and normal vitamin D groups if their serum 25-OH vitamin D levels were < 30 ng/mL and ≧ 30 ng/mL, respectively.

### Statistical analysis

All statistical analyses were carried out using the IBM SPSS for Windows software program version 22.0 (Armonk, NY: IBM Corp, USA). A p-value <0.05 was considered significant. Demographic and anthropometric data are expressed as mean ±standardized deviation (SD). Pearson correlation was adopted to explore the relationship between serum 25(OH)D and iPTH level. Chi-square test was used for determination of significance of difference between categorical variables. Multiple logistic regression analysis was performed to study the association between each presumed risk factor and vitamin D status, including age, gender, sun exposure, and vitamin D intake.

## Results

### Comparison of baseline characteristics between participants with low and normal serum vitamin D levels

**Fig 1:**
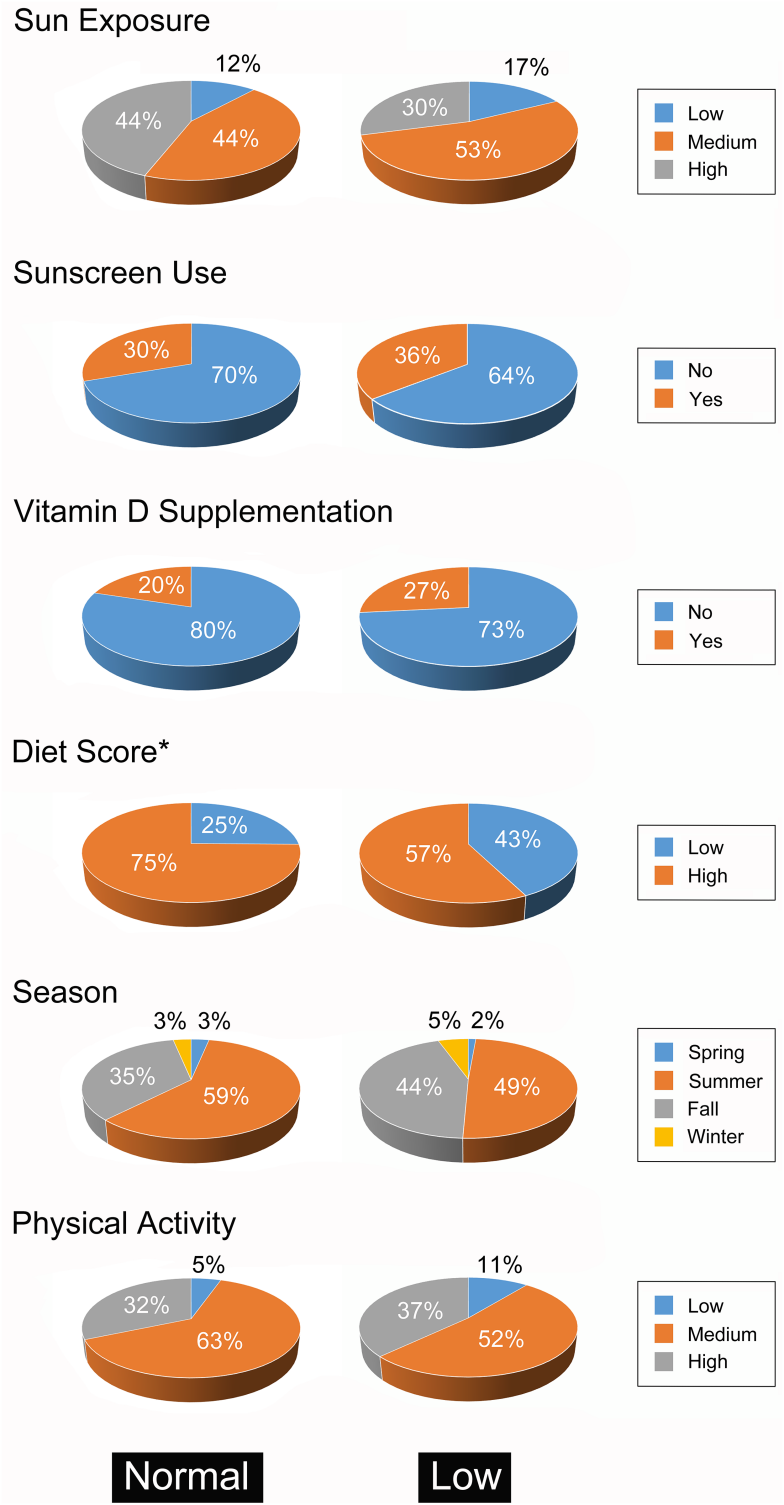
Comparison of factors affecting vitamin D level. Factors affecting vitamin D level between subjects (n = 170) with normal (n = 75) and low (n = 95) serum vitamin D level. *p <0.005 between the two groups (Chi-square test).

Totally 170 volunteers were recruited, including 85 men and 85 women with a mean age of 70.9 ± 5.6 years (range, 65–85) and a mean BMI of 24.0 ± 3.2 kg/m2 (range, 15.1–34.2) which was around the upper limit of normal (Table 1). Most participants had medium sun exposure (48.2%) without the habit of sunscreen use (67.1%) (Fig 1). In addition, most of them consumed adequate vitamin D through normal diets (67.1%) without the habit of taking vitamin D supplements (77.1%). According to the serum vitamin D levels, there were 75 (44.1%) and 95 (55.9%) testing subjects belonging to the low and normal vitamin D group, respectively. A low vitamin D status was demonstrated in 30.6% men and 57.7% of women. Gender was a significant indicator of vitamin D status. The majority of participants with low vitamin D status were women (65.3%) (*p* < 0.001). Dietary intake of vitamin D was another factor significantly associated with vitamin D status (*p* = 0.02). Age, BMI, sun exposure, use of sun screen, vitamin D supplement, season of blood withdrawal, or physical activity were not associated with vitamin D status.

**Table 1:** Comparison of demographic and anthropometric characteristics between low and normal vitamin D groups.

	Total (N = 170)	Low vitamin D group (N = 95) (25-OH vitamin D<30 ng/mL)	Normal vitamin D group (N = 75) (25-OH vitamin D≧30 ng/mL)	*P value
Age, years (mean ± SD)	70.9 ± 5.6	70.4 ± 5.7	71.2 ± 5.5	0.36
Gender				
Male, N (%)	85 (50%)	26 (34.7%)	59 (62.1%)	<0.001
Female, N (%)	85 (50%)	49 (65.3%)	36 (37.9%)	
Height, cm (mean ± SD)	159.5 ± 11.7	158.8 ± 15.4	160.0 ± 8.0	0.51
Weight, kg (mean ± SD)	60.9 ± 9.6	60.0 ± 9.7	61.7 ± 9.5	0.25
BMI, kg/m^2^ (mean ± SD)	24.0 ± 3.2	23.9 ± 3.4	24.1 ± 3.1	0.81

*Significance of difference determined by Chi-square test

### Correlations among serum vitamin D, calcium, and iPTH concentrations

**Fig 2:**
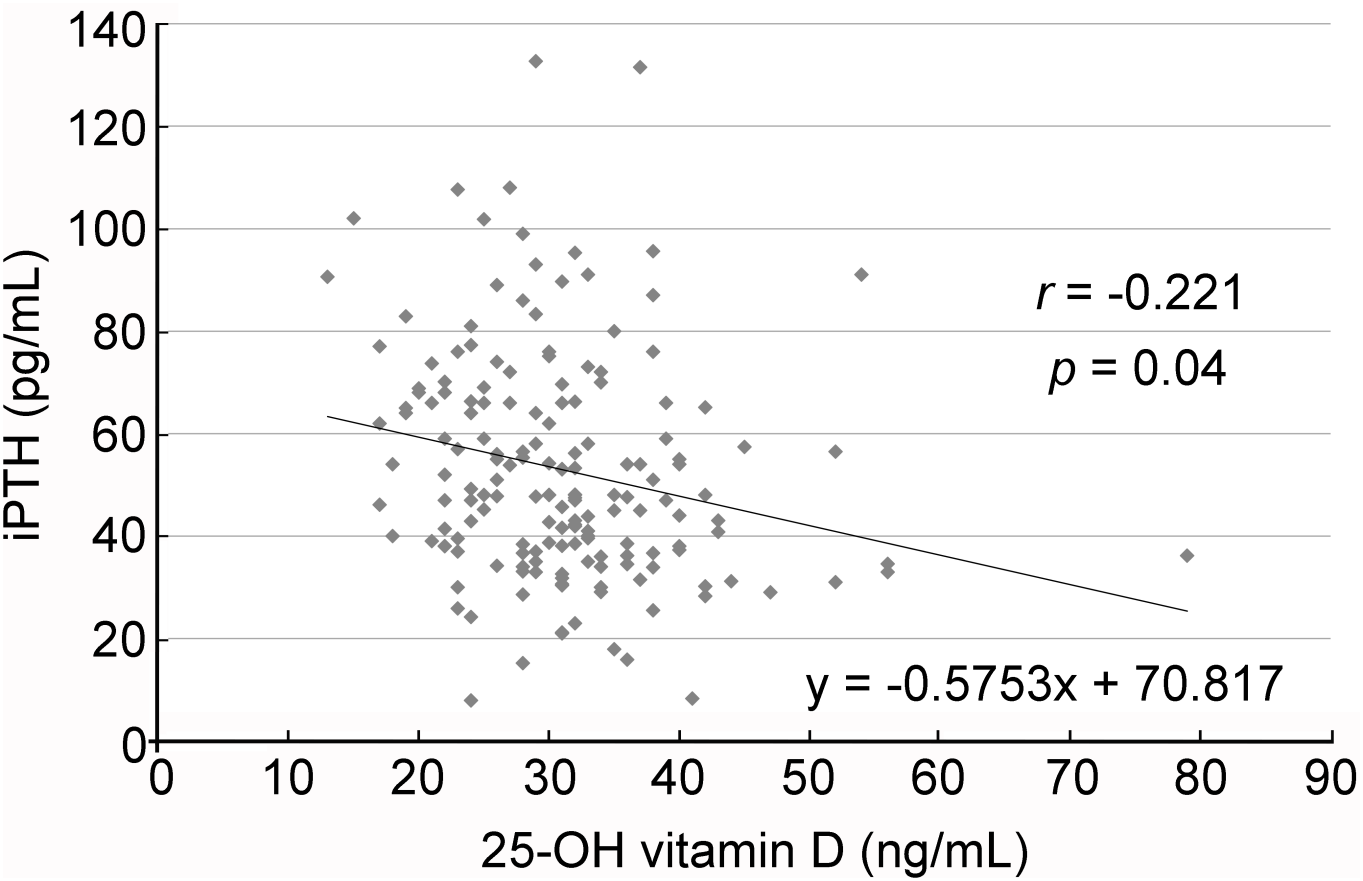
Relationship between serum parathyroid hormone (iPTH) concentration and 25-OH vitamin D level. Regression analysis with scatter plot showing inverse relationship between serum iPTH concentration and that of 25-OH vitamin D.

The participants showed a mean serum 25-OH vitamin D level of 30.9 ± 8.6 ng/mL, ranging from 13 to 79 ng/mL. On the other hand, their mean serum iPTH level was 53.0 ± 22.3 pg/mL, ranging from 7.9 to 132.7 pg/mL. Hyperparathyroidism was found in 21.76% of all participants (37/170) in whom the prevalence of hyperparathyroidism was higher in the low vitamin D group (29.33%, 22/75) than that in the normal vitamin D group (15.8%, 15/95). Scattered plot (Fig 2) showed a negative correlation between 25-OH vitamin D level and iPTH (r = -0.221, p = 0.004) after adjustment for age and sex. No significant correlation was noted between serum iPTH and serum calcium levels (r = 0.013, p = 0.865).

### Factor associated with low vitamin D status and gender differences

Chi-square test demonstrated significantly higher sun exposure in men than that in women (p = 0.003). The results of logistic regression of presumed risk factors for low vitamin D status were summarized in Table 2. Female gender was at higher risk of low vitamin D status (OR = 2.85, 95% CI: 1.39–5.85, p = 0.004) compared with that in their male counterparts. Low consumption of vitamin D-rich food was also significantly associated with an increased risk (OR = 2.09, 95% CI: 1.01–4.34, p = 0.049). In men (Table 3), inadequate intake of vitamin D-rich food was the only risk factor for a low vitamin D status (OR = 4.55, 95% CI: 1.43–14.43, p = 0.01, Nagelkerke r^2^ = 0.217). However, in women (Table 4), a lack of sun exposure was the only predictable risk with dose-response relationship. Compared with their female counterparts with high extent of sun exposure, elderly women with low sun exposure (OR = 6.84, 95% CI: 1.40–33.51, p = 0.018) and moderate sun exposure (OR = 6.67, 95% CI: 1.79–24.77, p = 0.005) were at greater risks of low vitamin D status (Nagelkerke r^2^ = 0.252).

**Table 2:** Logistic regression for risk factors of low vitamin D status.

	Odds ratio	95% CI		P value
Age	0.98	0.91	1.04	0.467
Gender				
Female	2.85	1.39	5.85	0.004
Male	1.00			
BMI	0.96	0.86	1.07	0.445
Sun exposure				
Low	1.39	0.46	4.19	0.561
Medium	1.71	0.79	3.70	0.176
High	1.00			
Sun screen				
No	1.28	0.58	2.84	0.541
Yes	1.00			
Vitamin D supplement				
No	0.75	0.32	1.75	0.504
Yes	1.00			
Diet score				
Low	2.09	1.01	4.34	0.049
High	1.00			
Season				
Spring	0.20	0.01	3.66	0.277
Summer	0.59	0.11	3.23	0.541
Fall	0.68	0.12	3.78	0.662
Winter	1.00			
Physical activity				
Low	1.14	0.27	4.77	0.859
Medium	0.53	0.25	1.11	0.092
High	1.00			

**Table 3:** Logistic regression for risk factors of low vitamin D status (Male).

	Odds ratio	95%CI		P value
Age	0.99	0.89	1.10	0.825
BMI	0.91	0.77	1.08	0.292
Sun exposure				
Low	0.24	0.02	2.98	0.264
Medium	0.57	0.18	1.80	0.335
High	1.00			
Sun screen				
No	0.91	0.26	3.16	0.884
Yes	1.00			
Vitamin D supplement				
No	0.73	0.18	3.03	0.663
Yes	1.00			
Diet score				
Low	4.55	1.43	14.43	0.010
High	1.00			
Season				
Spring	0.16	0.01	5.46	0.310
Summer	0.14	0.01	1.67	0.119
Fall	0.26	0.02	3.41	0.307
Winter	1.00			
Physical activity				
Low	0.47	0.44	0.05	4.125
Medium	0.53	0.70	0.22	2.160
High	1.00			

**Table 4:** Logistic regression for risk factors of low vitamin D status (Female).

	Odds ratio	95%CI		P value
Age	0.95	0.85	1.07	0.371
BMI	0.93	0.78	1.11	0.421
Sun exposure				
Low	6.84	1.40	33.51	0.018
Medium	6.67	1.79	24.77	0.005
High	1.00			
Sun screen				
No	1.78	0.52	6.03	0.357
Yes	1.00			
Vitamin D supplement				
No	1.00	0.29	3.44	1.000
Yes	1.00			
Diet score				
Low	1.16	0.40	3.33	0.788
High	1.00			
Season				
Spring	0.000	0.00		1.000
Summer	1.22	0.07	20.78	0.891
Fall	0.68	0.05	9.09	0.769
Winter	1.00			
Physical activity				
Low	2.88	0.23	36.01	0.412
Medium	0.65	0.19	2.24	0.498
High	1.00			

## Discussion

Although vitamin D deficiency and insufficiency are common worldwide, only a few studies have focused on the vitamin D status of the elderly. Moreover, limited surveys were completed in Asian countries so far. One multicenter survey showed a high prevalence of vitamin D deficiency in large cities in China, but no associated risk factors were reported [30]. Accordingly, our survey was designed to explore the risk factors of low vitamin D status in the elderly living in rural areas of Taiwan. Our survey demonstrated an incidence of low vitamin D status up to 44.1% in this population with female predominance (women: 57.7%, men: 30.6%).

Geographically, the percentage of vitamin D deficiency and insufficiency was higher than expectation in Asian countries, especially in South Asia [18]. In a multicenter vitamin D survey in China, the prevalence of vitamin D deficiency (<20 ng/mL) and vitamin D insufficiency (20–30 ng/mL) was 55.9% and 38.7%, respectively [30]. Another national survey in temperate areas of China reported the prevalence of vitamin D deficiency and insufficiency was 69.2 and 24.4%, respectively [25]. Based on the Korea National Health and Nutrition Examination Survey (KNHANES) 2008, Fourth Korea National Health and Nutrition Examination Surveys showed the prevalence of vitamin D deficiency was 47% in the males and 65% in the females [24]. As sun exposure is an essential source of vitamin D production, people living in low-latitude countries might be expected to have higher vitamin D levels than those living at high latitudes [1]. In contrast with China and Korea, Thailand, Taiwan and India are located at a lower latitude. However, the prevalence of low vitamin D status in people living in urban areas was reported to be up to 46.7% in one Thai study. For instance, the situation was more common in Bangkok (64.6%) than that in rural areas (33.5%), highlighting the impact of lifestyle and environmental factors on ultraviolet exposure and vitamin D synthesis [28]. In Taiwan, a survey including 215 participants living in Northern island demonstrated an incidence of 25-(OH)D deficiency up to 31% [34]. Even in tropical regions with plenty of sunshine such as India, more than 70% of the population suffered from vitamin D deficiency in all age groups and both genders [35].

Generally speaking, individual differences are significant contributors to vitamin D status. Vitamin D deficiency has been reported to be highly prevalent in pregnant women, children, older people, and residents of institutions [36]. Besides, more than 50% of postmenopausal women taking medication for osteoporosis also had 25-(OH)D ≤ 30ng/mL [37, 38]. Despite being a notorious culprit responsible for skin pigmentation, ultraviolet B triggers vitamin D production [16, 39]. Several studies have already revealed a lower vitamin D status in Asian women than that in their male counterparts [25, 40–42]. The finding may be explained by the observation that Asian women generally prefer indoor activities and take more precautions against sunlight exposure owing to aesthetic concern. The more frequent use of sunscreens in females than in males may also contribute to the difference [43]. Our research also identified the female gender as a risk factor for a low vitamin D status. Although use of sunscreen was not a predictive factor in our survey, the extent of sun exposure was shown to be positively associated with a high vitamin D status in females. In comparison to the high sun exposure group, the medium sun exposure group (OR = 6.67) and low sun exposure group (OR = 6.84) had significantly increased risks for low vitamin D status. On the other hand, the association was not shown among men. One reason may be that more men obtained sufficient sun exposure in contrast to women (92.94% v.s.78.83%), which implied the influence of sun exposure on vitamin D status of men was not as crucial as that of women.

Although exposure to sunlight for 5–30 minutes daily was considered adequate for the production of pre-vitamin D3 in adults, old age is a significant predictor of low vitamin D status regardless of seasons or the extent of sun exposure [1, 44]. Indeed, based on skin biopsy samples, the capacity of production of pre-vitamin D3 in the elderly was less than half of that in young adults [26]. Besides, it has been reported that a 70-year-old person only produced one-fourth of the amount of vitamin D compared with that of a 20-year-old through cutaneous synthesis [27]. This may partly account for the high prevalence of low vitamin D status in the rural areas of Taiwan, which is located in the subtropical region. Although the present study showed that the majority of the elderly population (85.89%) had adequate sun exposure because of farming, gardening, and other outdoor activities, they were not exempted from vitamin D sufficiency. The finding, therefore, may suggest a more detrimental influence of age on vitamin D status compared to that of inadequate sun exposure.

Consumption of vitamin D-rich food, such as fortified milk, meat, oily fish, eggs, has been demonstrated to be limited in the diets of adolescents, regardless of races and genders [45–47]. Similarly, it has been reported that the elderly seldom take adequate daily amount to meet recommended daily allowance [1]. Moreover, for the aged population, in addition to the reduced absorption of dietary vitamin D associated with aging, polypharmacy commonly seen has also been shown to reduce absorption of vitamin D because of drug-food interaction [48]. Therefore, the importance of vitamin D supplementation increases by age. In our logistic regression model, only elderly men benefited from vitamin D-rich food to maintain adequate vitamin D status. The odds ratio of having a low vitamin D status in men with low diet score was 4.55 times higher than that in those with a high score. However, our results failed to show significant association between diet habits and serum vitamin D level among women. One possible explanation is that, unlike the case in men, factors other than diet such as menopausal status, lifestyle, and environmental influence may play a more important role in determining vitamin D status in women. [23, 49]

Predictors of vitamin D status were introduced in several large cohort studies, such as race, gender, body mass index, residential area, vitamin D supplement, dietary intake, and season [50, 51]. These possible indicators were all included in our logistic regression. In addition, gender was divided in subgroups to explore the difference between male and female living in the rural areas. Some previous studies, but not all, showed gender differences consistent with our results [52] [53]. Our data further revealed gender differences in predictors of low vitamin D status in the elderly. Moreover, the present study demonstrated that inadequate sun exposure and insufficient dietary vitamin D intake were significantly associated with low vitamin D status in women and men, respectively. The association between a low vitamin D status and malignancy has also been reported. Previous reviews [54, 55] showed a significant inverse relationship between colorectal cancer and vitamin D level, while the association in other malignancies including breast cancer, prostate cancer are less well defined [56, 57]. Another Asian study also supported the inverse relationship between vitamin D level and risk of rectal cancer [58]. In addition, previous epidemiological studies also showed that vitamin D level was negatively associated with cardiovascular risks, although benefit of vitamin D supplementation was not demonstrated in interventional studies [59]. On the other hand, another study reported an association between a low vitamin D status and the prevalence of osteoporosis, which was shown to improve with oral vitamin D supplementation [60]. The importance of gender influence on vitamin D status in the elderly as well as the role of vitamin D in the prevention and therapy of cancer, osteoporosis, cardiovascular diseases warrant further exploration [56, 61, 62].

Regarding the role of vitamin D in specific Taiwanese patient populations, there have been two studies investigating the impact of vitamin D level on disease progression. One of the studies investigated the influence of vitamin D status on bone complications in post-menopausal women [63], while the other studied the clinical significance of vitamin D level in patients with chronic hepatitis B [64]. The study focusing on vitamin D status and osteoporosis in 199 middle-aged to elderly postmenopausal women demonstrated a prevalence of vitamin D inadequacy (i.e., insufficiency and deficiency) of 86.6% [63]. Moreover, the other study on relatively young patients aged around 37 with chronic hepatitis B revealed a prevalence of vitamin D insufficiency and deficiency of 35% and 58%, respectively, both of which were shown to adversely affect prognostic outcome [64]. The two studies, therefore, highlight the high prevalence of vitamin D inadequacy in the diseased populations regardless of age and gender.

There are some limitations in the current study. Firstly, relatively young individuals were not recruited in the present study for evaluation of the impact of age on vitamin D status. Secondly, only subjects in rural areas were studied so that the vitamin D status of their counterparts in urban areas was unavailable for comparison. Thirdly, the information on some predictors that cannot be accurately quantitated, including dietary intake of vitamin D and sun exposure, was obtained through questionnaires that may have caused reporting and recall bias. Finally, the cross-sectional design of this study precluded the investigation of causative effects of demographic and lifestyle factors on vitamin D status. Further cohort studies, therefore, are warranted to address these issues.

## Conclusions

In conclusion, low vitamin D status, including vitamin D insufficiency and deficiency, was common in the aged population living in subtropical rural areas. Low sun exposure and inadequate dietary vitamin D consumption were associated with a status of low vitamin D in females and males, respectively.

## Supporting information

S1 Table Food frequency questionnaire (FFQ). English version of FFQ estimating oral vitamin D intake through assessing the frequency of vitamin D-rich food consumption in recent 3 months.
(DOCX)

S2 Table Food frequency questionnaire (FFQ). Original Chinese version of FFQ estimating oral vitamin D intake through assessing the frequency of vitamin D-rich food consumption in recent 3 months.
(DOCX)

S3 Table Vitamin D contents of vitamin D-rich food items included in the food frequency questionnaire (FFQ). Vitamin D contents of commonly available vitamin D-rich food items included in FFQ. IU: International unit.
(DOCX)
