# Supplementary material for: Prevalence and predictors of hypovitaminosis D among the elderly in subtropical region
Source: PLoS One. 2017 Jul 31;12(7):e0181063. doi: 10.1371/journal.pone.0181063 (PMC5536299; doi:10.1371/journal.pone.0181063)
Supplement: S1 Table — English version of FFQ estimating oral vitamin D intake through assessing the frequency of vitamin D-rich food consumption in recent 3 months. (DOCX) [file pone.0181063.s001.docx]

**Supporting Information 1**

**S1 Table. Food frequency questionnaire (FFQ).** English version of FFQ estimating oral vitamin D intake through assessing the frequency of vitamin D-rich food consumption in recent 3 months.

Food frequency questionnaire (FFQ)

In recent 3 months, how many times per week do you eat the following foods?

1. Raw fish or oysters □Never □1-2 times □>=3 times
2. Cooked fish (e.g. eel, sardines, tuna, salmon, mackerel, herring, including Canned fish) □Never □1-2 times □>=3 times
3. Eggs □Never □1-2 times □>=3 times
4. Vitamin D-fortified cereals □Never □1-2 times □>=3 times
5. Dairy foods that may have been fortified with vitamin D (e.g. cheese, fortified soymilk, and fortified milk) □Never □1-2 times □>=3 times

Total scores: ___________ (Never: 0 point; 1-2 times: 1 point; >=3 times: 2 points)
