# Supplementary material for: Prevalence and predictors of hypovitaminosis D among the elderly in subtropical region
Source: PLoS One. 2017 Jul 31;12(7):e0181063. doi: 10.1371/journal.pone.0181063 (PMC5536299; doi:10.1371/journal.pone.0181063)
Supplement: S2 Table — Original Chinese version of FFQ estimating oral vitamin D intake through assessing the frequency of vitamin D-rich food consumption in recent 3 months. (DOCX) [file pone.0181063.s002.docx]

**Supporting Information 2**

**S2 Table. Food frequency questionnaire (FFQ).** Original Chinese version of FFQ estimating oral vitamin D intake through assessing the frequency of vitamin D-rich food consumption in recent 3 months.

飲食調查問卷

最近三個月內，平均每週食用以下食物頻率為何?

1. 生魚片或牡蠣 □未食用 □1-2次 □3次以上 (包含3次)
2. 煮熟鹹水魚類(鰻魚、沙丁魚、鯖魚、鮪魚、鮭魚等，包含鯖魚罐頭或油漬鮪魚罐頭) ^1^□未食用 □1-2次 □3次以上 (包含3次)
3. 雞蛋 □未食用 □1-2次 □3次以上 (包含3次)
4. 維生素D強化穀類 □未食用 □1-2次 □3次以上 (包含3次)
5. 維生素D強化乳製品 □未食用 □1-2次 □3次以上 (包含3次)

總分:___________ (從未: 0分; 1-2次: 1分; >=3次: 2分)
