# Supplementary material for: Prevalence and predictors of hypovitaminosis D among the elderly in subtropical region
Source: PLoS One. 2017 Jul 31;12(7):e0181063. doi: 10.1371/journal.pone.0181063 (PMC5536299; doi:10.1371/journal.pone.0181063)
Supplement: S3 Table — Vitamin D contents of commonly available vitamin D-rich food items included in FFQ. IU: International unit. (DOCX) [file pone.0181063.s003.docx]

**Supporting Information 3**

**S3 Table. Vitamin D** **contents of vitamin D-rich food items included in the food frequency questionnaire (FFQ).** Vitamin D contents of commonly available vitamin D-rich food items included in FFQ. IU: International unit.

| Food Item | Vitamin D content |
| --- | --- |
| Raw fish or oysters | 600-1200 IU/100g |
| Cooked fish (e.g. eel, sardines, tuna, salmon, mackerel, herring, including Canned fish) | 250-300 IU/100g |
| Eggs | 20–50 IU/yolk |
| Vitamin D-fortified cereals | 60–80 IU/100 mL |
| Dairy foods that may have been fortified with vitamin D (e.g. cheese, fortified soymilk, and fortified milk) | 7–28 IU/ 100 g |
